# Relationship between carotid intima-media thickness and white matter hyperintensities in non-stroke adults: a systematic review

**DOI:** 10.3389/fnana.2024.1394766

**Published:** 2024-06-06

**Authors:** Syeda Humayra, Noorazrul Yahya, Chai Jia Ning, Mohd Asyiq Al-Fard bin Mohd Raffali, Imtiyaz Ali Mir, Abdul Latiff Mohamed, Hanani Abdul Manan

**Affiliations:** ^1^Makmal Pemprosesan Imej Kefungsian (Functional Image Processing Laboratory), Department of Radiology, University Kebangsaan Malaysia, Kuala Lumpur, Malaysia; ^2^Diagnostic Imaging & Radiotherapy Program, School of Diagnostic & Applied Health Sciences, Faculty of Health Sciences, Universiti Kebangsaan Malaysia, Kuala Lumpur, Malaysia; ^3^Department of Radiology and Intervention, Hospital Pakar Kanak-Kanak (UKM Specialist Children’s Hospital), Universiti Kebangsaan Malaysia, Malaysia; ^4^Cardiology Unit, Department of Medicine, Hospital Canselor Tuanku Muhriz, Universiti Kebangsaan Malaysia, Bandar Tun Razak, Malaysia; ^5^Department of Physiotherapy, M Kandiah Faculty of Medicine and Health Sciences, Universiti Tunku Abdul Rahman, Kajang, Malaysia; ^6^Faculty of Medicine, University of Cyberjaya, Cyberjaya, Malaysia

**Keywords:** carotid atherosclerosis, carotid intima-media thickness, stroke, white matter hyperintensity, systematic review

## Abstract

**Introduction:**

Literature suggests a common pathophysiological ground between carotid atherosclerosis (CAS) and white matter alterations in the brain. However, the association between carotid intima-media thickness (CIMT) and white matter hyperintensities (WMH) has not been conclusively reported. The current systematic review explores and reports the relationship between CIMT and WMH among asymptomatic/non-stroke adults.

**Methods:**

A recent literature search on PubMed, SCOPUS, and Web of Science databases was conducted in compliance with the PRISMA protocol. The pre-defined Population-Intervention-Comparison-Outcome-Study (PICOS) criteria included observational studies investigating the CIMT-WMH association among non-stroke adults undergoing magnetic resonance imaging and carotid ultrasound.

**Results:**

Out of 255 potential results, 32 studies were critically assessed for selection, and finally, 10 articles were included, comprising 5,116 patients (females = 60.2%; males = 39.8%) aged between 36–71 years. The included studies earned high quality ratings (6–9) based on the Newcastle-Ottawa-Scale criteria. Qualitative synthesis showed a significantly parallel relationship between increased CIMT and greater WMH burden in 50% of the studies. In addition, significant risk factors related to the CIMT-WMH association included older age, hypertension, depression, migraine, Hispanic ethnicity, and apolipoprotein E (ɛ4) in postmenopausal women.

**Conclusion:**

Overall, the cumulative evidence showed a consistent CIMT-WMH association in asymptomatic middle-aged and older non-stroke adults, indicating that CAS may contribute to the progression of pathologically hyperintense white matter in the brain. However, further research is warranted to infer the plausible relationship between CIMT and WMH in the absence of stroke.

## Introduction

Elevated carotid intima-media thickness (CIMT) and cerebral white matter hyperintensities (WMH) of presumed vascular origin serve as indicators of large vessel disease (LVD) and small vessel disease (SVD), respectively. Although they result from damage in distinct vascular regions and stem from different pathological processes, they may still manifest concurrently (Del Brutto et al., 2023). CIMT is a useful marker in measuring the extent of carotid atherosclerosis (CAS) among asymptomatic patients, and emerging evidence suggests a strong link between CAS and WMH, mainly due to insufficiency in cerebral perfusion (Zhang et al., 2021).

CIMT is a quantitative assessment of the inner layers of the carotid artery walls using B-mode ultrasound, encompassing the intima (inner layer) and media (middle layer). This frequently utilized cardiovascular disease (CVD) predictor has been linked to several risk factors such as age, hypertension, dyslipidaemia, and smoking (Tschiderer et al., 2020). In contrast, WMHs also referred to as leukoaraiosis or white matter lesions, are pathological regions of heightened signal intensity that are best visible on T2-weighted (T2w) magnetic resonance imaging (MRI) scans. These hyperintense lesions of varying sizes are frequently located in the cerebral white matter of the brain and are often associated with SVD, ischemia, and neurodegenerative disorders (Parent et al., 2023). Although the precise aetiology of WMH is not well comprehended, yet its prevalence tends to elevate with older age, female sex, hypertension, cardiac diseases, type 2 diabetes, obesity, hyperlipidaemia, smoking, alcohol abuse, and renal impairment (Sun et al., 2022). The cerebrovascular disease burden, assessed by the total WMH volume, is being highly recognized for its significant contribution towards the cognitive manifestations in Alzheimer’s disease (AD) (Parent et al., 2023), and increased risk of vascular dementia, ischaemic stroke, and death (Ye et al., 2018).

Literature indicates there may be a shared underlying pathophysiological mechanism between carotid artery disease and white matter abnormalities in the brain (Liao et al., 2015). Despite the mounting evidence suggesting a possible connection between CAS and WMH, there remains a controversial debate (Liao et al., 2015). Hence, deciphering the CIMT-WMH relationship is crucial in the field of vascular and neurological research, since they are associated with various clinical conditions including cognitive decline, AD, and increased risk of future stroke outcomes (Ye et al., 2018). Previously systematic reviews have focused on the relationship between CIMT and cognitive decline (Fresnais et al., 2021), emphasized the carotid stenosis severity and white matter subtypes (Ye et al., 2018), and included symptomatic/stroke patients, who may be susceptible to pre-existing white matter alterations (Liao et al., 2015). Therefore, it can be inferred that the CIMT-WMH relationship has been earlier reported with inconclusive findings and thus necessitates a further meticulous investigation. Keeping this in mind, the current review paper aims to address the existing research gap and answer two key research questions: (1) Is there a relationship between carotid intima-media thickness and white matter hyperintensities in non-stroke adults? (2) What are the related factors or risk predictors of the CIMT-WMH association?

## Methodology

A systematic review of the literature was conducted to examine the association between CIMT and WMH in asymptomatic adults showing no signs or history of stroke. In accordance with the preferred reporting items for systematic reviews and meta-analysis (PRISMA) (Liberati, 2009) and previous study guidelines (Manan et al., 2020; Voon et al., 2021; Yap et al., 2022; Sahrizan et al., 2023; Yahya and Manan, 2023), two researchers independently conducted an extensive search on PubMed, SCOPUS, and Web of Science databases supplemented by a manual search of the included articles’ reference lists. The search was restricted to the English language and included only original research articles published in the last decade (February 2014–January 2024). To identify potentially relevant articles, we used the broad search terms “carotid intima-media thickness” and “white matter.”

**Figure 1:**
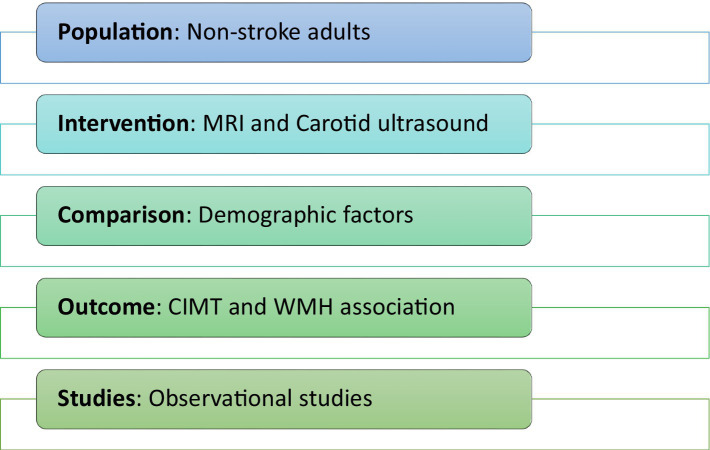
PICOS criteria for selection of eligibility.

Two reviewers evaluated the eligibility requirements and independently examined titles and abstracts for inclusion after removing duplicate literature. This was followed by assessing full-text records based on the selection criteria. Studies were considered eligible if they met the pre-specified PICOS criteria illustrated in Figure 1. Inclusion criteria for the studies were: (i) observational studies; (ii) with measurements of carotid intima-media thickness and white matter hyperintensity; (iii) where all subjects underwent scanning procedures such as MRI and carotid ultrasound; (iv) the full-text was accessible; (v) publication time frame between 2014–2024; (vi) English language; (vii) and involved human subjects. Exclusion criteria included patients with a history of stroke, transient ischaemic attack, and cerebrovascular diseases. Papers in which the association between WMH and CIMT was unclear/missing or could not be identified were excluded. White matter changes resulting from non-vascular causes were also excluded. The decision to include or exclude studies was conducted independently by two reviewers, with disagreements resolved by mutual discussion. In addition, two reviewers individually assessed the quality of the included studies using the Newcastle-Ottawa Scale (NOS). This scale defines a study with six or more stars as high-quality (Margulis et al., 2014). Any disagreements were resolved through consensus.

Two researchers independently assembled information from the included articles using a structured table and cross-checked the data for further accuracy. The general attributes of the studies were collected, such as the first author’s name, publication year, country, study design and setting, number of patients involved, patient demographics including age and gender distribution, ethnicity and education along with clinical characteristics such as BMI, systolic and diastolic blood pressure, antihypertensives usage and smoking history. In addition, the CIMT and WMH measures, risk factors and outcomes were also recorded. A third reviewer verified all extracted data, and disagreements were resolved by consensus. There was no communication with the study authors for any undisclosed or missing data. Due to heterogeneity in the study populations and outcomes, a meta-analysis was not performed.

Mean (SD) and median (IQR) values of CIMT, and WMH percentiles or its median values as proportion of the whole brain volume/intracranial volume were extracted. CIMT assessment followed the consensus recommendations and involved obtaining measurements in a region without plaque and recording readings from distinct locations such as CCA, carotid bulb, and internal carotid artery separately (Fernández-Alvarez et al., 2022). Normal carotid intima-media thickness values for healthy middle-aged individuals typically range from 0.6 to 0.7 mm, while CIMT measurements exceeding 0.90 mm are suggestive of organ damage or an indication of absolute coronary artery disease risk according to the European Society of Cardiology guidelines (Mohamed et al., 2023). WMH was assessed through quantitative volumetric measurement and/or visual categorization method using the Fazekas (Fz) scores ranging from 0–3 to quantify the severity of WMH, as both are validated approaches in clinical practice (Andere et al., 2022). The primary study outcome was the CIMT and WMH association in the included studies. Whereas the secondary outcomes were the patient characteristics and/or clinical markers associated with CIMT-WMH. This review has been registered publicly on the Open Science Framework research platform (doi: 10.17605/OSF.IO/Q8V76).

## Results

**Figure 2:**
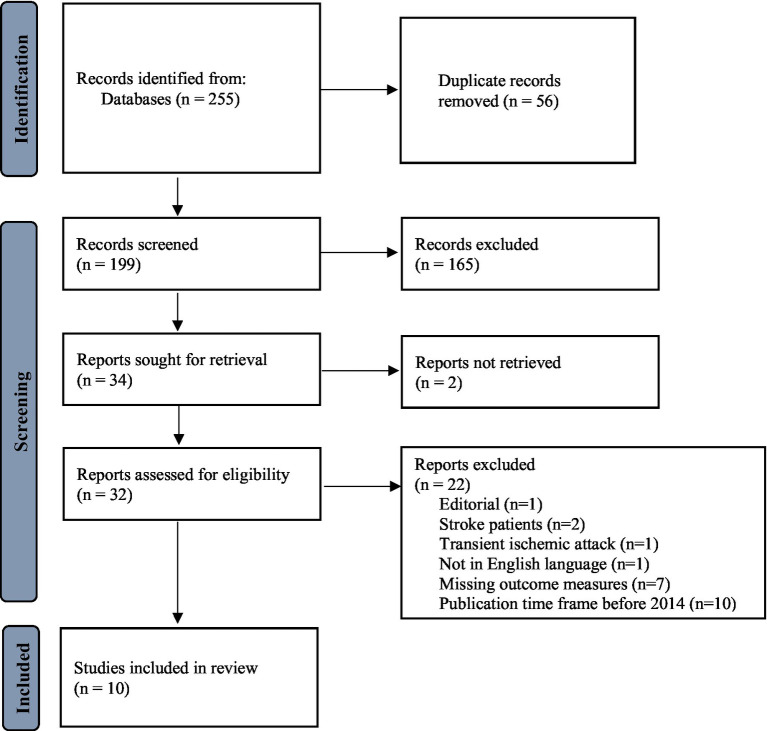
Flowchart of study selection.

Out of 255 potential results on an electronic search, 32 studies were critically assessed for selection, and finally, 10 were included in this systematic review. The search and selection process and reasons for exclusion have been outlined in Figure 2.

A total of ten studies comprising 5,116 patients with 60.2% females and 39.8% males were included in the qualitative analysis. The majority of the studies were carried out in the USA (Della-Morte et al., 2018; Cermakova et al., 2020; Wang et al., 2022; Thurston et al., 2023) and China (Chen et al., 2019; Zhang et al., 2020; Zheng et al., 2020), while others were in Finland (Inkeri et al., 2021), Turkey (Kocatürk and Kocatürk, 2020), and Denmark (Devantier et al., 2016). Nearly all studies adopted a cross-sectional design; except one, which was a case-control study (Devantier et al., 2016). Four studies were carried out in a community setting (Della-Morte et al., 2018; Zhang et al., 2020; Wang et al., 2022; Thurston et al., 2023), four in a university-based hospital (Devantier et al., 2016; Chen et al., 2019; Kocatürk and Kocatürk, 2020; Zheng et al., 2020), and two in a multicentric setting (Cermakova et al., 2020; Inkeri et al., 2021). Although the study populations largely varied, most of the studies reported no history of CVD events and/or cognitive impairment. The studies’ sample sizes ranged from 29 to 1795. All studies administered a T2w fluid-attenuated inversion recovery (FLAIR) MRI sequence imaging. Seven out of ten studies utilized a high-resolution 3 T MRI scan, while others used a 1.5 T field strength (Della-Morte et al., 2018; Chen et al., 2019; Kocatürk and Kocatürk, 2020). Most of the studies measured specific regional readings of the intima-media thickness involving both internal and common carotid arteries, except one study that measured the CCA-IMT only (Zheng et al., 2020). The majority of the studies utilized volumetric imaging measurements of hyperintense white matter, and three studies used semi-quantitative methods with Fz score ≥ 1 (Inkeri et al., 2021) and ≥2 (Chen et al., 2019; Zhang et al., 2020). The NOS scores ranged between 6 to 9 which indicated an overall high quality of the included studies. Table 1 shows the characteristics of the included studies published between 2016 to 2023.

**Table 1:** Study characteristics.

Study author	Year of publication	Country	Study design	Setting	Study population	Clinical characteristics	*n* (patient/control)	Imaging intervention		Outcome measures		Study quality (NOS)
Thurston et al.	2023	USA	Cross-sectional	Community	Middle-aged women (perimenopausal/postmenopausal)	No CVD events Healthy cognition	224	Carotid ultrasound (B-mode)	MRI (3 T, FLAIR)	CIMT (carotid arteries)	WMHV (WBV)	9
Wang et al.	2022	USA	Cross-sectional	Community	Atherosclerosis at-risk	No CVD events Healthy cognition	1795	Carotid ultrasound (B-mode)	MRI (3 T, FLAIR)	CIMT quintiles (carotid arteries)	WMHV (total ICV)	8
Inkeri et al.	2021	Finland	Cross-sectional	Multicenter	Neurologically asymptomatic Type-1 Diabetic/HCs	NR	186/30	Carotid ultrasound	MRI (3 T, FLAIR)	CIMT (carotid arteries)	WMH (Fazekas score ≥ 1)	7
Kocatürk et al.	2020	Turkey	Cross-sectional	University hospital	Migraine without aura/HCs without migraine	No CVD events Healthy cognition	75/30	Carotid ultrasound (Doppler)	MRI (1.5 T, FLAIR)	CIMT (carotid arteries)	WMH	6
Zhang et al.	2020	China	Cross-sectional	Community	Cerebral small vessel disease in rural population	No CVD events	904	Carotid ultrasound	MRI (3 T, FLAIR)	CIMT (carotid arteries)	WMH (Fazekas score ≥ 2)	8
Zheng et al.	2020	China	Cross-sectional	Medical college hospital	Dialysis patients	No CVD events	73	Carotid ultrasound	MRI (3 T, FLAIR)	CIMT (common carotid only)	WMH (WBV)	6
Cermakova et al.	2020	USA	Cross-sectional	Multicenter	Biracial middle-aged	NR	461	Carotid ultrasound	MRI (3 T, FLAIR)	CIMT (carotid arteries)	WMHV (WBV)	7
Chen et al.	2019	China	Cross-sectional	University hospital	Hypertensive patients	Healthy cognition	140	Carotid ultrasound	MRI (1.5 T, FLAIR)	CIMT (carotid arteries)	WMH (Fazekas score ≥ 2)	8
Della-Morte et al.	2017	USA	Cross-sectional	Community	Multiethnic older adults	NR	1,229	Carotid ultrasound	MRI (1.5 T, FLAIR)	CIMT (carotid arteries)	WMHV (total ICV)	8
Devantier et al.	2016	Denmark	Case-control	University hospital	Late-onset major depressive disorder/HCs without depression	NR	29/27	Carotid ultrasound (B-mode)	MRI (3 T, FLAIR)	CIMT (carotid arteries)	WMHV (WBV)	6

CIMT, carotid intima-media thickness; CVD, cardiovascular disease; FLAIR, fluid-attenuated inversion recovery; HCs, healthy controls; ICV, intracranial volume; MRI, magnetic resonance imaging; NR, not reported; PV, periventricular; MHV, white matter hyperintensity volume; NOS, Newcastle-Ottawa-Scale; WBV, whole brain volume; WML, white matter lesion.

The subjects’ age ranged between 36–71 years, and about 70% of the included studies had patients above 55 years. Across the studies, the majority of the patients were of female sex (*n* = 3,080), Black (*n* = 816), and Hispanic ethnicity (*n* = 799). Approximately, 50% of the studies reported individuals with higher secondary education. In terms of the WMH lesion category, deep white matter hyperintensity (DWMH) was more prevalent in the subjects than periventricular white matter hyperintensity (PWMH), since the area of WMH localization was mostly reported within the deep cerebral white matter. The patients’ systolic blood pressure ranged from 116.9 to 140.3 mmHg and their body mass index (BMI) ranged between 22.9 to 28.8 kg/m^2^. The majority of the studies reported smokers (80%) and individuals on antihypertensive medications (70%) as shown in Table 2.

**Table 2:** Patient characteristics.

Study	Age	Sex *n* (%)	Race/Ethnicity *n* (%)	Education	WMH localization	Apolipoprotein g/L	BMI kg/m^2^	Blood Pressure mmHg	Use of AntiHTNs *n* (%)	Smoking *n* (%)
Thurston et al. (2023)	59.19 (4.27)	Females only	Non-Hispanic White = 184 (82.14%), Black = 29 (12.95%) Asian/mixed = 11 (4.91%)	Years of education = 15.75 (2.37)	Deep white matter and in the occipital lobe	APOE = 48 (22.43%)	26.71 (23.79, 31.83)	SBP = 118.37 (14.14) DBP = 68.26 (8.84)	Yes AntiHTNs = 40 (17.86%)	NR
Wang et al. (2022)	57.0 (6)	*F* = 1,089 (60.7%)	Black = 412 (23.0%)	Less than high school = 251 (14%)	Deep cerebral white matter	APOE = 512 (28.5%)	28.0 (4.8)	SBP = 119.4 (16.0)	Yes AntiHTNs = 385 (21.4%)	Yes 271 (15.1%)
Inkeri et al. (2021)	40.0 (33.0–45.0)	*F* = 97 (52.2%)	NR	NR	NR	APOB = 67.0 (59.0–83.0)	26.2 (24.0–28.7)	SBP = 129.3 (119.0–139.5)	Yes	NR
Kocatürk and Kocatürk (2020)	36 (30–40)	*F* = 60 (80.0%)	NR	NR	Frontal = 30 (90.9%) Deep = 29 (87.9%)	NR	24.6 (22.27–26.72)	NR	Yes	Yes 14 (18.7%)
Zhang et al. (2020)	59.7 (3.0)	*F* = 500 (55.3%)	NR	NR	PV and deep regions	NR	24.2 (3.2)	SBP = 137.7 (20.1) DBP = 82.4 (11.8)	Yes AntiHTNs = 272 (30.1%)	Yes 284 (31.6%)
Zheng et al. (2020)	62.4 (9.6)	*F* = 46 (63.0%)	NR	More than high school = 15 (37.5%)	NR	NR	22.9 (3.6)	SBP = 140.3 (17.8) DBP = 76.2 (11.0)	Yes	Yes 16 (21.9%)
Cermakova et al. (2020)	50.6 (3.4)	*F* = 250 (54.0%)	African Americans = 158 (34.0%)	Years of education = 15.2 (2.3)	NR	NR	28.3 (5.4)	SBP = 116.9 (13.6) DBP = 72.8 (10.6)	NR	Yes 63 (14.0%)
Chen et al. (2019)	69.2 (10.5)	*F* = 64 (45.7%)	NR	NR	PV and deep regions	NR	28.8 (4.2)	SBP = 131.7 (16.3) DBP = 75.8 (9.9)	Yes AntiHTNs = 105 (75.0%)	Yes 37 (26.4%)
Della-Morte et al. (2018)	71 (9)	*F* = 737 (60.0%)	Hispanic = 799 (65.0%), Black = 217 (17.7%), White = 184 (15.0%)	NR	NR	NR	28 (5)	NR	NR	Yes 116 (9.4%)
Devantier et al. (2016)	59.8 (4.5)	*F* = 13 (45.0%)	NR	Years of education = 14.4 (2.7)	NR	NR	26.6 (3.7)	NR	NR	Yes

APO, apolipoprotein; AntiHTNs, antihypertensives; BMI, body mass index; DBP, diastolic blood pressure; F, females; SBP, systolic blood pressure.

The highest CIMT value (0.90 mm) was found among patients with SVD in a rural population (Zhang et al., 2020) and the lowest (0.48 mm) was reported in migraine patients (Kocatürk and Kocatürk, 2020). WMH percentiles in proportion to the total brain volume ranged from 16.7% (Kocatürk and Kocatürk, 2020) to 79.0% (Cermakova et al., 2020), and the median volumes ranged between 0.06 (Wang et al., 2022) to 1.25 (Devantier et al., 2016). A significant CIMT-WMH association (Table 3) was deduced in 50% of the included studies, where five studies reported a directly proportional relationship (Devantier et al., 2016; Della-Morte et al., 2018; Chen et al., 2019; Kocatürk and Kocatürk, 2020; Thurston et al., 2023), indicating an increased CIMT coexists with heightened WMH burden. It was observed that CIMT did not correlate significantly with WMH volume in the biracial middle-aged population (Cermakova et al., 2020), those at risk of atherosclerosis (Wang et al., 2022), dialysis patients (Zheng et al., 2020), cerebral SVD (Zhang et al., 2020), and type-I diabetes (Inkeri et al., 2021).

**Table 3:** Association between carotid intima-media thickness and white matter hyperintensity.

Study	CIMT (mean/median)	WMH (median/frequency)	Findings
Thurston et al. (2023)	0.77 (0.31)	0.065 (0.043, 0.102)	Higher CIMT was significantly associated with greater whole brain and periventricular WMHV
Wang et al. (2022)	>0.80	0.06 (−0.08, 0.20)	No significant association between CIMT and WMHV
Inkeri et al. (2021)	0.58 (0.55–0.65)	31 (16.7%)	Arterial stiffness and CIMT were increased in subjects with type 1 diabetes with WMHs, however, it did not correlate significantly
Kocatürk and Kocatürk (2020)	0.48 (0.44–0.53)	33 (44.0%)	CIMT was significantly greater in migraine patients with WMH
Zhang et al. (2020)	0.9 (0.3)	172 (19.0%)	Presence of carotid plaque was associated with WMHV, but no significant relationship between CIMT and WMHV on independent multivariate analysis
Zheng et al. (2020)	1.2 (1.0–1.4)	25 (34.2%)	No significant difference between presence of WMH in normal and increased CIMT groups
Cermakova et al. (2020)	0.71 (0.15)	361 (79.0%)	CIMT was not significantly associated with WMH or the brain volume
Chen et al. (2019)	0.77 (0.19)	46 (33.0%)	Higher WMH grade was associated with increased CIMT
Della-Morte et al. (2018)	0.71 (0.08)	0.36 (0.21–0.76)	Significant relationship between increased CIMT and increased brain WMHV. CCA diameter was a significant and independent contributor to WMHV burden
Devantier et al. (2016)	0.91 (0.8–1.2)	1.25 (1.94–3.06)	An increase in averaged CIMT of 0.1 mm was associated with 52% increase in WMHV

CCA, common carotid artery; CIMT, carotid intima-media thickness; WMHV, white matter hyperintensity volume.

**Figure 3:**
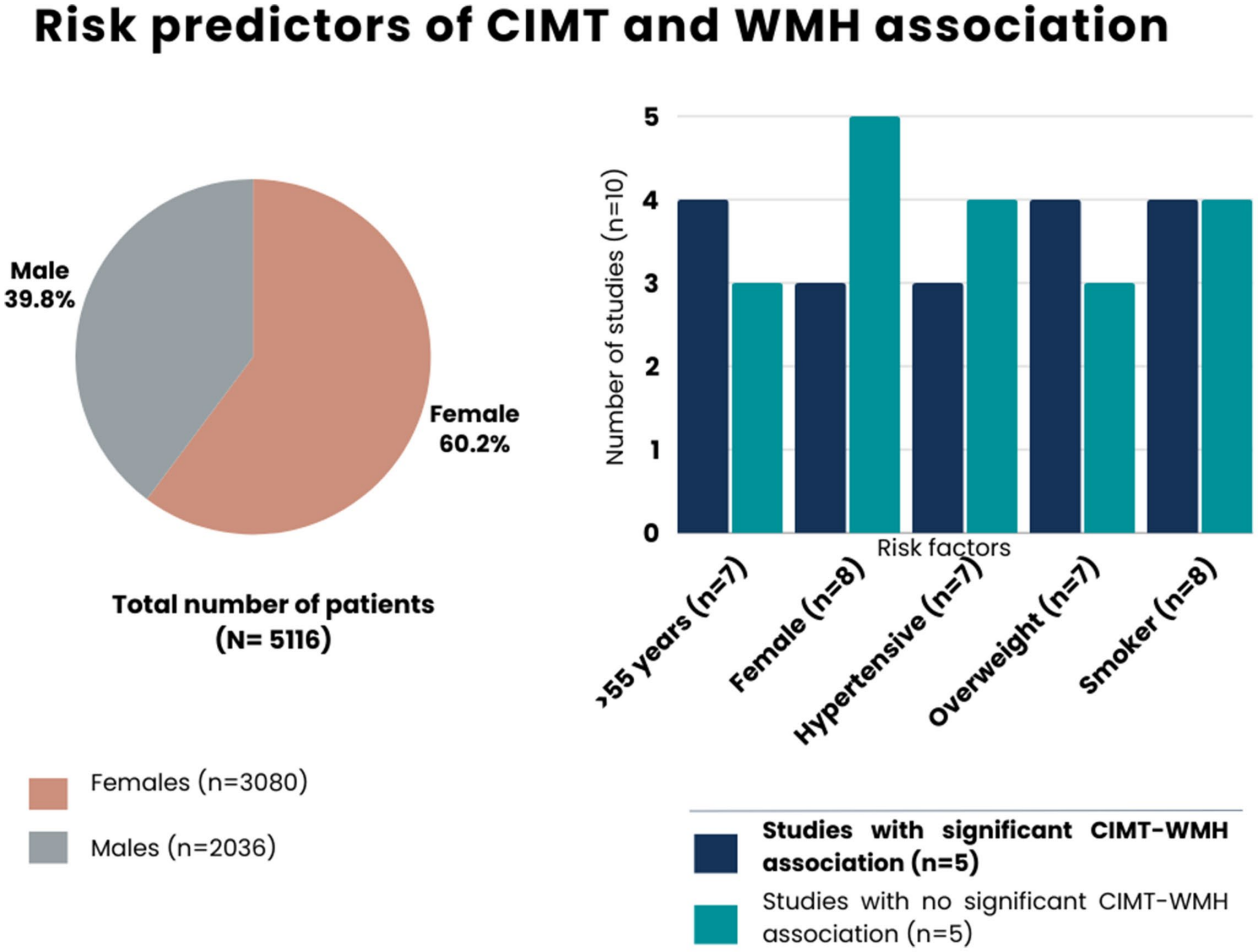
Risk predictors of carotid intima-media thickness and white matter hyperintensity.

In terms of demographic and clinical factors, females, elderly and hypertensive adults, smokers, and overweight people (BMI >25 kg/m^2^) were highly prevalent across the studies but older age and high BMI were considerably greater in studies showing significant CIMT-WMH relationship (Figure 3). In addition, significant risk factors related to the CIMT-WMH association (Table 4) included older age (Della-Morte et al., 2018), migraine and depressed patients with WMH (Kocatürk and Kocatürk, 2020), hypertension (Chen et al., 2019), Hispanic ethnicity (Della-Morte et al., 2018), and the presence of apolipoprotein E (APOE) ɛ4 allele in postmenopausal women (Thurston et al., 2023).

**Table 4:** Risk factors and outcomes related to increased CIMT and WMH burden.

Study	Risk factors	Outcomes
Thurston et al. (2023)	APOE ɛ4 carriers	Higher CIMT associated with greater WMHV particularly for APOEε4–positive middle-aged women
Wang et al. (2022)	Older, males, chain smokers, hypertension, and lower education status	Increased midlife CIMT and presence of carotid plaque associated with smaller deep GMV and cortical volume in a temporal-parietal meta ROI
Inkeri et al. (2021)	Individuals with type 1 diabetes with the presence of CMBs	Higher CIMT was associated with CMBs, independent of age, APOB, and SBP
Kocatürk and Kocatürk (2020)	Migraine patients with presence of WMH	CIMT was higher in migraine patients with WMH
Zhang et al. (2020)	Hypertensive population and females	Increased CIMT correlated with a higher LAC and PVS
Zheng et al. (2020)	Increased CIMT group was older and had lower serum albumin and creatinine levels	Demonstrated a gradual decrease in the brain-matter volume and degenerated cognitive function
Cermakova et al. (2020)	Lower CBF in middle age was related to higher atherosclerotic risk	Increased CIMT was associated with lower CBF in gray matter and the total brain
Chen et al. (2019)	Hypertensive patients with increased ambulatory systolic blood pressure	24-h SBP, 24-h SBP-SD, and CIMT were independently associated with WMH burden
Della-Morte et al. (2018)	Older age and Hispanic ethnicity	Greater CIMT was associated with higher cerebral WMH burden among elderly and Hispanic individuals
Devantier et al. (2016)	Depressed patients	A significantly higher WMH load was observed in depressed patients which associated with CAS

APO, apolipoprotein; CAS, carotid atherosclerosis; CBF, cerebral blood flow; CIMT, carotid intima-media thickness; CMBs, cerebral microbleeds; GMV, grey matter volume; LAC, lacunes; SBP, systolic blood pressure; SD, standard deviation; PVS, perivascular spaces; ROI, region of interest; WMH, white matter hyperintensity.

## Discussion

The current review aimed to synthesize the available evidence and provide a comprehensive understanding of the relationship between carotid intima-media thickness and white matter hyperintensities in non-stroke adults, including the potential risk factors, clinical implications, and areas for future research. The aggregated qualitative analysis showed that regardless of the different patient populations, a significantly parallel association between increased CIMT and greater WMH burden was noted in 50% of the studies, which comprised 33.2% of the total patient population; however, other half of the studies with approximately 66.8% patients found no significant relationship.

WMH is a significant public health issue that raises the risk of dementia and stroke and has been increasingly linked to LVDs such as carotid atherosclerosis (Liao et al., 2015). As a potential preclinical marker for atherosclerotic load and endothelial impairment, CIMT is often utilized to assess the risk and progression of both cardiovascular and cerebrovascular disorders (Ou et al., 2023). The burden of vascular dementia and cognitive dysfunction is substantially mediated by aging, which imposes a tremendous risk upon the middle-aged and older populations (Del Brutto et al., 2020). In addition, the combined effects of the large cerebral arteries involving CAS and SVD are most likely connected to the age- and hypertension-related increases in the magnitude and severity of WMHs (Ye et al., 2018). The overall prevalence of WMH in young individuals (31–45 years) is approximately 25.94%, which tends to rise with increasing age (Zheng et al., 2023). A recent study in China found that age and hypertension were independently associated with an increased WMH prevalence in older, stroke-free population, aged 60 years and above (Zheng et al., 2023). This is consistent with the current findings, whereby a majority of our study population were older hypertensive adults. Similarly, in relatively young and healthy populations without any conventional cardiovascular risk factors, a CIMT less than 0.8 mm is considered typical, and their progression to carotid plaques is about 7% (Limbu et al., 2015). In our study, a higher percentage of female patients with increased CIMT and WMH were reported, although the findings were not statistically different. Since women experience a crucial reproductive transition after menopause, they may tend to exhibit an increased intima-media thickness buildup, stiffness, and vascular remodeling (Thurston et al., 2023).

CAS may be connected to WMH since the carotid artery is a major blood vessel that supplies blood to the brain. Possible pathological mechanisms linking CAS to white matter load, include the classic CVD risk factors, such as elevated blood pressure, increased obesity, greater insulin resistance, and altered lipid profiles that are related to both greater CIMT and WMH volume (Thurston et al., 2023). Vascular risk factors may potentially worsen athero-inflammation state, compromise the integrity of the blood–brain barrier (BBB), and promote neuroinflammation in people who have not experienced a stroke, possibly preparing the brain for future damage (Evans et al., 2021). Thus, long-term pro-inflammatory conditions like atherosclerosis might be accountable for the BBB integrity being compromised. Hence this kind of BBB impairment could be related to the development of white matter alterations and marked by the loss of neurons, demyelination, and gliosis (Evans et al., 2021). The high prevalence and clinical importance of WMH is garnering greater attention with breakthroughs in brain imaging technologies. As the population ages, the prevalence of WMH load consequentially increases the risk of dementia, impaired mobility, and stroke (Cermakova et al., 2020; Thurston et al., 2023). This is because age is an independent risk factor for both CIMT and WMH. Therefore, identifying the modifiable risk factors related to CIMT-WMH prevalence in middle-aged and older adults, its prevention and treatment modalities is crucial due to its significant impact on patients and the healthcare system.

The possible explanation for studies with no significant CIMT and WMH correlation might be due to the severity/degree of carotid stenosis, which was probably inadequate to cause hemodynamic changes involving lowered cerebral blood flow and leading toward the development of hyperintense white matter lesions (Ye et al., 2018). Secondly, two of those studies comprised of individuals with comparatively younger age and normal intima-media thickness measures (Cermakova et al., 2020; Inkeri et al., 2021). Another study that reported an increased CIMT with a significant reduction in the brain white matter among patients with chronic kidney disease might be due to the dialysis-induced cerebral blood flow changes resulting in brain atrophy (Zheng et al., 2020).

This study comes with some notable strengths as well as limitations, which should be taken into consideration. Our study has systematically articulated recently published data and contributed to the scientific repository on CIMT-WMH association in non-stroke patients for the first time. An earlier systematic review included most patients with symptomatic ischemic stroke and measured WMH using visual rating scales instead of quantitative volumetric measurements, which was a major limitation due to its shortened sensitivity and accuracy (Liao et al., 2015). Furthermore, the included studies in their review reported heterogeneous imaging techniques for assessing the WMH burden and CAS which might have influenced the study findings by limiting or overestimating it (Liao et al., 2015). In contrast, our study mostly included papers that used high-resolution MRI with automated quantitative volumetric methods for measuring WMH, and carotid duplex scan to demonstrate the increase in CIMT. The definition and analysis of the plaque data in CIMT studies have varied widely (Naqvi and Lee, 2014). This might have led to different definitions of the severity of carotid stenosis and its inclusion criteria in past studies ranging from 30–50%, thus contributing to heterogeneity and inconsistency in results (Ye et al., 2018). At the same time, due to the duplex ultrasound scan’s limited accuracy in identifying mild stenosis, there is a greater possibility of bias in measuring carotid stenosis (Liao et al., 2015). The distinction between increased CIMT and plaque is arbitrary, and disagreement exists over whether carotid intima-media thickness and plaques are specific phenotypic entities or a transitional pathway from increased CIMT to plaque formation (Naqvi and Lee, 2014). A similar issue was faced in this review because there were certain differences in the CIMT definitions and their quantification methods and sites based on the scanners’ waveforms and sonographers’ protocol. Certain data inconsistencies were noted, in terms of the patient’s cognitive function, cardiovascular history, and ethnic background which could have influenced the study findings. Patient recruiting times to running scans have varied across the studies, in terms of time interval between the carotid ultrasound (measurement of CIMT) and brain MRI (measurement of WMH). Since our review aimed to examine the CIMT-WMH association in the absence of stroke, studies conducted among patients with history of stroke, transient ischaemic attack, and cerebrovascular diseases had to be excluded to minimize the possibility of pre-existing brain alterations including white matter changes which resulted in a relatively small number of eligible articles for inclusion. Lastly, due to the cross-sectional nature of most studies, causality cannot be drawn from the given inferences. The findings of this comprehensive analysis should be evaluated with consideration due to the potential variations in the study population and setting, which may affect its general applicability.

Some of the studies highlighted the interaction of genetic apolipoprotein variants in conjunction with WMH burden (Inkeri et al., 2021; Wang et al., 2022; Thurston et al., 2023), and found that higher CIMT was linked to greater WMH volume particularly in APOEε4-positive postmenopausal women (Thurston et al., 2023). In addition, a significantly higher WMH load in depressed patients (Devantier et al., 2016), and increased CIMT in migraine patients with WMH was observed (Kocatürk and Kocatürk, 2020). Therefore, timely intervention programs targeting the at-risk populations in clinical settings, along with middle-aged and older CVD patients should be highly prioritized for neuroimaging markers and cognitive decline. Simultaneously, smoking cessation, weight loss with a nutritious diet plan, and optimal blood pressure regulation measures should be administered by healthcare professionals for effective management. Understanding the relationship between CIMT and WMH in non-stroke adults may provide valuable insights into the underlying vascular and neurologic processes that contribute to the development of both conditions. Further research is warranted to elucidate the exact underlying mechanisms and potential causal relationship between CIMT and WMH in the absence of stroke. Additionally, investigating this relationship can have clinical implications for risk stratification, early detection, and prevention strategies for both cardiovascular and cerebrovascular diseases.

## Conclusion

Across the included studies, the coexistence of CIMT and WMH was observed, however, a significant relationship was attained in 50% of the studies. This systematic review revealed a consistent CIMT-WMH association among asymptomatic middle-aged and older non-stroke adults, suggesting that CAS may contribute to the development or progression of white matter abnormalities in the brain. These findings highlight the importance of monitoring carotid health in preclinical settings as a potential precursor for white matter pathology and necessitate an early intervention plan.

## Data availability statement

The original contributions presented in the study are included in the article/Supplementary material, further inquiries can be directed to the corresponding author.

## Author contributions

SH: Conceptualization, Data curation, Formal analysis, Investigation, Methodology, Visualization, Writing – original draft, Writing – review & editing. NY: Data curation, Formal analysis, Investigation, Validation, Writing – review & editing, Methodology. CN: Data curation, Formal analysis, Investigation, Validation, Writing – review & editing, Methodology. MR: Data curation, Formal analysis, Investigation, Validation, Writing – review & editing. IM: Data curation, Formal analysis, Methodology, Validation, Writing – review & editing. AM: Formal analysis, Investigation, Methodology, Validation, Writing – review & editing. HM: Conceptualization, Formal analysis, Investigation, Supervision, Validation, Writing – review & editing, Methodology.
